# Assessment of vitamin D among male adolescents and young adults hospitalized with eating disorders

**DOI:** 10.1186/s40337-022-00627-5

**Published:** 2022-07-18

**Authors:** Jason M. Nagata, Anna Grandis, Paola Bojorquez-Ramirez, Anthony Nguyen, Amanda E. Downey, Kyle T. Ganson, Khushi P. Patel, Vanessa I. Machen, Sara M. Buckelew, Andrea K. Garber

**Affiliations:** 1 Department of Pediatrics, University of California, 550 16th Street, 4th Floor, Box 0110, San Francisco, CA 94143 USA; 2 Yale School of Public Health, Yale University, New Haven, CT 06510 USA; 3 Department of Psychiatry and Behavioral Sciences, University of California, San Francisco, San Francisco, CA 94143 USA; 4 Factor-Inwentash Faculty of Social Work, University of Toronto, Toronto, ON Canada

**Keywords:** Feeding and eating disorders, Male, Boys, Vitamin D, Adolescent

## Abstract

**Purpose:**

Medical complications of eating disorders in males are understudied compared to females, as is the case of vitamin D deficiency. The aim of this study was to assess vitamin D levels among male and female adolescents and young adults hospitalized for medical complications of eating disorders.

**Methods:**

We retrospectively reviewed electronic medical records of patients aged 9–25 years (N = 565) admitted to the University of California, San Francisco Eating Disorders Program for medical instability, between May 2012 and August 2020. Serum vitamin D (25-hydroxy) level was assessed at admission as was history of prior calcium, vitamin D, or multivitamin supplementation. Linear regression was used to assess factors associated with vitamin D levels.

**Results:**

A total of 93 males and 472 females met eligibility criteria (age 15.5 ± 2.8, 58.8% anorexia nervosa; admission body mass index 17.6 ± 2.91). Among male participants, 44.1% had 25-hydroxyvitamin D levels < 30 ng/mL, 18.3% had 25-hydroxyvitamin D levels < 20 ng/mL, and 8.6% had 25-hydroxyvitamin D levels < 12 ng/mL. There were no significant differences in 25-hydroxyvitamin D levels in males compared to females, except that a lower proportion (1.9%) of female participants had 25-hydroxyvitamin D levels < 12 ng/mL (*p* = 0.001). Only 3.2% of males reported calcium or vitamin D-specific supplementation prior to hospital admission, while 8.6% reported taking multivitamins. White race, prior calcium/vitamin D supplementation, and higher calcium levels were associated with higher vitamin D levels on admission.

**Conclusions:**

Nearly half of patients admitted to the hospital for malnutrition secondary to eating disorders presented with low 25-hydroxyvitamin D levels; males were more likely than females to have severe vitamin D deficiency. These findings support vitamin D assessment as part of the routine medical/nutritional evaluation for hospitalized eating disorder patients, with particular attention on male populations.

**Supplementary Information:**

The online version contains supplementary material available at 10.1186/s40337-022-00627-5.

## Plain English summary

Medical complications of eating disorders in males are understudied compared to females. Little is known about vitamin D levels in male adolescents and young adults with eating disorders. We studied vitamin D levels in male and female adolescents and young adults hospitalized at the University of California, San Francisco between 2012 and 2020. We found that 44% of males had low vitamin D levels. Despite nearly half of males having low vitamin D, few were receiving calcium, vitamin D, or multivitamin supplementation prior to the hospitalization. White race, prior calcium or vitamin D supplementation, and higher calcium levels were associated with higher vitamin D levels. Medical providers should consider assessing for low vitamin D levels among males and females with eating disorders.

**Supplementary Information:**

The online version contains supplementary material available at 10.1186/s40337-022-00627-5.

## Introduction

Eating disorders are psychiatric diagnoses characterized by binging or restricting of nutritional intake, fear of gaining weight, and disturbances in body image, with a high prevalence in adolescents and young adults [1]. Eating disorders have one of the highest mortality rates of psychiatric disorders due to associated medical and psychiatric complications. Many of these complications are secondary to malnutrition, with the majority of patients having at least one micronutrient deficiency [2]. Vitamin D is the second most prevalent micronutrient deficiency in hospitalized patients with eating disorders (after zinc deficiency), with more than half of patients having low serum vitamin D [2]. Vitamin D deficiency can yield inadequate bone growth and mineralization, and emerging research underscores its crucial role in neurodevelopment [3]. For instance, lower vitamin D levels are associated with higher risk-taking and impulsivity in patients with eating disorders [4]. Moreover, adolescence and young adulthood is a critical period for growth and development [5–8], where macro- and micronutrient deficiencies can be particularly detrimental.

Males with eating disorders are understudied compared to females, despite increasing recognition of their prevalence among adolescents with eating disorders [9–14]. Previous studies of adolescents with eating disorders have illustrated vitamin D deficiency and its ramifications including low bone mineral density and osteoporosis [1, 15], though other factors (e.g., inadequate nutrition, low body mass index, hypogonadism, low growth hormone) may also contribute to low bone density [16, 17]. However, the number of male participants in these studies was small, ranging from n = 3 to n = 14 [1–3]; thus, differences in eating disorder presentation and severity were not disaggregated between sexes. This crucial gap in knowledge demands further investigation with robust male inclusivity to inform sex-specific guidelines.

Unique eating disorder behaviors among males may contribute to the false perception that they are not malnourished. Such behaviors may include increased protein intake and muscle-building supplements to increase body mass, as opposed to the caloric restriction and drive for thinness typically seen in females [9, 11, 18]. Vitamin D is a fat-soluble vitamin which is distributed into fat and muscle tissue [19], and may be sequestered in adipose tissue [20]. Greater fat and muscle mass is associated with low serum vitamin D, likely secondary to a volumetric dilution of serum vitamin D to these other body stores [19, 21]. Therefore, higher protein intake and muscle-building exercises could also result in low serum levels of vitamin D compared to restricting behaviors [22, 23]. On the other hand, patients with restrictive eating disorders often reduce dietary fat intake [24]; dietary fat can facilitate vitamin D absorption [25]. This preference for low fat intake has mostly been studied in females with eating disorders [24], though males with eating disorders may also restrict fat intake [18]. Recent studies demonstrate no sex differences in fat mass when adjusting for confounders such as degree of malnourishment in adolescents with anorexia nervosa (AN) [26]. Males with eating disorders experience skeletal complications including bone fractures [27]. Deficits in bone mineral density are equally severe in adolescent males and females with AN [28]. We hypothesized that vitamin D deficiency is equally severe in males as in females with EDs. Therefore, the objective of this study was to determine vitamin D levels in male adolescents and young adults hospitalized with eating disorders.

## Methods

### Study population

Data were collected from the electronic medical records (EMR) of 601 patients presenting for inpatient hospitalization due to medical instability to the Eating Disorders Program at the University of California, San Francisco (UCSF) between May 2012 and August 2020. Patients were between the ages of 9–25 years at admission. An eating disorder diagnosis was made by a psychologist or psychiatrist for the purposes of clinical care. Although a minority of patients (7.8%) were initially diagnosed using DSM-IV criteria (prior to May 2013), we reviewed their clinical and psychosocial characteristics and recategorized them using DSM-5 criteria. For instance, we reviewed the charts of participants with a DSM-IV diagnosis of Eating Disorder Not Otherwise Specified (EDNOS) and reclassified them into an appropriate DSM-5 eating disorder diagnosis. Evaluation by the medical team includes an assessment of the history (including medical history) and presenting symptoms, physical examination, and laboratory tests (comprehensive metabolic panel, inflammatory markers, liver function, thyroid studies, amylase, uric acid, a complete blood count, cholesterol, and others) to evaluate for other etiologies of malnutrition and/or weight loss such as malabsorption and metabolic disorders. Additional evaluation and specialist consultation is pursued as appropriate.

We included patients between the ages of 9–25 years with an initial hospitalization for the medical management of an eating disorder between May 22, 2012 and August 31, 2020. Patients with missing vitamin D measurements were excluded from the study (n = 36); the final analytic sample consisted of 565 patients.

### Study design

Per inpatient protocols, sociodemographic data, anthropometric measurements, disease characteristics, and lab data were documented in the EMR for each participant [29, 30]. Clinical assessments in the EMR were retrospectively reviewed and entered into the UCSF Eating Disorders Program Medical Database. Body mass index (BMI, kg/m^2^) was calculated using initial weight and height measurements on admission. Height was measured on a wall-mounted stadiometer. Weights were obtained each morning by standardized protocol with the patient in a hospital gown without undergarments on a standing scale prior to any nutritional intake and immediately post-void. Percent median BMI (%mBMI) was calculated using calculated BMI and median BMI for age and sex [31]. Vitamin D (25-hydroxy vitamin D) and a comprehensive metabolic panel (including calcium and phosphorous) were collected at the time of admission per routine admission protocol and before treatment with medications or nutritional supplements. All samples were drawn using a standardized lab protocol and analyzed at a Clinical Laboratory Improvement Amendments (CLIA)-certified laboratory, which follows federal standards and regulations. 25-hydroxyvitamin D level was analyzed using chemiluminescent microparticle immunoassay (Abbott Architect i2000 and ci4100). Consensus on the classification of vitamin D sufficiency, insufficiency, and deficiency remains debated. The Endocrine Society [32] and Society for Adolescent Health and Medicine [33] define vitamin D insufficiency as 20–30 ng/mL and deficiency as < 20 ng/mL. The Institute of Medicine defines deficiency as < 12 ng/mL [34, 35]. These definitions were incorporated in our study to create binary variables using three thresholds (insufficiency < 30 ng/mL, deficiency < 20 ng/mL, and severe deficiency < 12 ng/mL). We created a seasonality variable based on the date of the 25-hydroxy vitamin D lab test given that the effect of season on vitamin D level has been well documented in North America, with serum vitamin D reaching a nadir in winter months [36]. We dichotomized the seasons as has been done previously in Northern California [37] (summer [April through September] vs. winter [October through March]) based on average hours of sunshine in San Francisco [38].

### Ethics

This retrospective chart review study involving human participants was in accordance with the ethical standards of the institutional and national research committee and with the 1964 Helsinki Declaration and its later amendments or comparable ethical standards. The Institutional Review Board (IRB) of the University of California, San Francisco approved this study (20–30323). A waiver of informed consent was approved given no more than minimal risk to participants, the study will not adversely affect the rights and welfare of participants, the retrospective chart review could not practicably be done without the waiver, and an adequate plan to protect the identifiers from improper use and disclosure.

### Statistical analysis

Data analysis was conducted using Stata 15.1 (StataCorp LP, College Station, TX). We compared demographic and clinical characteristics of patients who were included versus excluded in Additional file 1: Table 1 using independent samples t-tests for continuous variables (age, BMI), Chi-squared tests for categorical variables (race/ethnicity, diagnosis), and Fisher’s exact tests for categorical variables with five or fewer participants in a cell (sex). To provide baseline comparisons between male and female groups with respect to demographic characteristics, BMI variables, and laboratory values, we used independent samples t-tests, Chi-squared tests, or Fisher’s exact tests. Simple linear regression analyses were used to examine the association between demographics and 25-hydroxyvitamin D levels. For the purposes of the regression, we dichotomized race/ethnicity (non-Hispanic White vs all other race/ethnicities) and diagnosis (anorexia nervosa vs. all other diagnoses) given small numbers of participants in the other categories. All variables significantly associated with 25-hydroxyvitamin D levels in unadjusted analyses were entered into a multivariable model with 25-hydroxyvitamin D level as the dependent variable. Sensitivity analyses were conducted excluding participants with active calcium or vitamin D supplementation immediately prior to admission.

## Results

This cross-sectional study included 93 male and 472 female adolescent and young adult participants after excluding 4 male and 32 female participants due to missing 25-hydroxyvitamin D data (Additional file 1: Table 1). There were not significant differences in sex, age, race/ethnicity, diagnosis, or BMI for those included versus excluded; however, non-Hispanic white participants were more likely to be included compared to other races/ethnicities. Demographic and clinical characteristics by sex are shown in Table 1. The average age was 15.5 years and mean %mBMI was 87.0%. Among male participants, 44.1% had 25-hydroxyvitamin D levels < 30 ng/mL, 18.3% had 25-hydroxyvitamin D levels < 20 ng/mL, and 8.6% had 25-hydroxyvitamin D levels < 12 ng/mL. By comparison, only 1.9% of female participants had 25-hydroxyvitamin D levels < 12 ng/mL (p = 0.001). Mean calcium level was slightly lower in males than in females (9.3 vs 9.5, p = 0.005) while mean phosphorous levels were similar. In a sensitivity analysis excluding participants with active calcium or vitamin D supplementation immediately prior to admission, findings were similar although the rates of 25-hydroxyvitamin D levels < 30 ng/mL (45.6%), < 20 ng/mL (18.9%), and < 12 ng/mL (8.9%) in males were slightly higher (Additional file 1: Table 2).

**Table 1:** Demographic characteristics and nutritional status of adolescents and young adults hospitalized for restrictive eating disorders by sex

Characteristic	Total (N = 565)	Sex		p ^a^
		Male (n = 93)	Female (n = 472)	
Age, years, mean (sd)	15.49 ± 2.80	15.71 ± 2.67	15.44 ± 2.83	0.409
Race/ethnicity, n (%)				**0.015**
Non-Hispanic white	343 (60.71)	46 (49.46)	297 (62.92)	
Hispanic	94 (16.64)	27 (29.03)	67 (14.19)	
Asian or NHOPI^b^	44 (7.79)	5 (5.38)	39 (8.26)	
Multiracial	28 (4.96)	5 (5.38)	23 (4.87)	
Other	28 (4.96)	3 (3.23)	25 (5.30)	
Unknown/Declined	15 (2.65)	2 (2.15)	13 (2.75)	
Non-Hispanic Black or African American	13 (2.30)	5 (5.38)	8 (1.69)	
Diagnosis, n (%)				**0.005**
Anorexia nervosa	332 (58.76)	40 (43.01)	292 (61.86)	
Unspecified feeding and eating disorder (UFED)	55 (9.73)	9 (9.68)	46 (9.75)	
Other	44 (7.79)	11 (11.83)	33 (6.99)	
Avoidant restrictive food intake disorder (ARFID)	32 (5.66)	12 (12.90)	20 (4.24)	
Other specified feeding and eating disorder (OSFED)	92 (16.28)	20 (21.51)	72 (15.25)	
Bulimia nervosa	9 (1.59)	1 (1.08)	8 (1.69)	
Binge-eating disorder	1 (0.18)	0	1 (0.21)	
BMI, kg/m^2^, mean (sd)	17.55 ± 2.91	17.84 ± 3.77	17.49 ± 2.72	0.296
% median BMI, mean (sd)	87.00 ± 14.00	87.03 ± 17.48	87.00 ± 13.23	0.987
25-hydroxyvitamin D (ng/mL), mean (sd)	32.15 ± 11.87	30.06 ± 11.69	32.57 ± 11.87	0.063
25-hydroxyvitamin D categories, n (%)				
Insufficiency (< 30 ng/mL)	243 (43.01)	41 (44.09)	202 (42.80)	0.818
Deficiency (< 20 ng/mL)	70 (12.39)	17 (18.28)	53 (11.23)	0.059
Severe deficiency (< 12 ng/mL)	17 (3.01)	8 (8.60)	9 (1.91)	**0.001**
Month (season) of Vitamin D lab, n (%)				0.346
Summer (April through September)	309 (54.69)	55 (59.14)	254 (53.81)	
Winter (October through March)	256 (45.31)	38 (40.86)	218 (46.19)	
Calcium, serum/plasma (mg/dL), mean (sd)	9.42 ± 0.45	9.31 ± 0.62	9.45 ± 0.41	**0.005**
Phosphorous, serum/plasma (mg/dL), mean (sd)	3.98 ± 1.90	3.99 ± 0.66	3.98 ± 2.06	0.987
Any Calcium, vitamin D, or multivitamin supplementation, n (%)	89 (15.75)	10 (10.75)	79 (16.74)	0.148
Calcium or vitamin D specific supplementation, n (%)	46 (8.14)	3 (3.23)	43 (9.11)	0.062
Vitamin D supplementation	29 (5.13)	3 (3.23)	26 (5.51)	0.362
Calcium supplementation	31 (5.49)	2 (2.15)	29 (6.14)	0.122
Multivitamin supplementation, n (%)	64 (11.33)	8 (8.60)	56 (11.86)	0.364

Boldface indicates *p* < 0.05

^a^*P*-value is for t-tests for continuous variables or Pearson's chi square tests (or Fisher's exact test as appropriate) for categorical variables, respectively. For race/ethnicity, p-value is for Pearson's chi square test comparing a binary race/ethnicity variable (non-Hispanic white vs all other race/ethnicities) by sex (male vs female). For diagnosis, p-value is for Pearson's chi square test comparing a binary diagnosis variable (anorexia nervosa vs all other diagnoses) by sex (male vs female)

^b^NHOPI = Native Hawaiian and Other Pacific Islanders

Only 3.2% of males reported receiving calcium or vitamin D-specific supplementation prior to the hospital admission, while 8.6% reported taking multivitamins. Overall, 10.8% of males reported taking any calcium, vitamin D, or multivitamin supplementation prior to hospital admission, compared to 16.7% of females.

In unadjusted linear regression analyses, lower percent median BMI, White race, higher serum calcium, summer, prior calcium/vitamin D and multivitamin supplementation prior to admission was associated with higher 25-hydroxyvitamin D levels (Table 2). Sex, age, eating disorder diagnosis (anorexia nervosa vs non-anorexia nervosa), and serum/plasma phosphorous were not significantly associated with 25-hydroxyvitamin D levels. Adjusted linear regression analyses included percent median BMI, White race, prior calcium/vitamin D supplementation, prior multivitamin supplementation, summer, and calcium level as independent variables. In the adjusted model, White race, prior calcium/vitamin D supplementation, summer, and calcium were associated with higher 25-hydroxyvitamin D level. Specifically, White race was associated with 7.63 (95% CI 5.75, 9.51) higher serum 25-hydroxyvitamin D level compared to non-White race. Each one unit (mg/dL) higher serum/plasma calcium level was associated with a 2.19 (0.14, 4.23) higher 25-hydroxyvitamin D level (ng/mL). Findings were similar in a sensitivity analysis excluding participants with active calcium or vitamin D supplementation immediately prior to admission, except that anorexia nervosa (compared to other diagnoses) was associated with higher 25-hydroxyvitamin D levels in unadjusted but not adjusted analyses and summer (compared to winter) was associated with higher 25-hydroxyvitamin D levels in adjusted but not unadjusted analyses (Additional file 1: Table 3).

**Table 2:** Factors associated with 25-hydroxyvitamin D level, adjusted linear regression analysis

Independent variables	Unadjusted		Adjusted	
	B (95% CI)^a^	*P*	B (95% CI)^b^	*p*
Male sex	−2.51 (−5.15, 0.13)	0.063	–	–
Age, years	0.01 (−0.34, 0.36)	0.973	–	–
White (vs. non-White)	**7.99 (6.09, 9.88)**	** < 0.001**	**7.63 (5.75, 9.51)**	** < 0.001**
Anorexia nervosa (vs. non-anorexia nervosa)	1.91 (−0.07, −3.90)	0.059	–	–
Percent median BMI	−**7.22 (**−**14.21, **−**0.23)**	**0.043**	−4.02 (−10.58, 2.53)	0.228
Summer (vs. winter)	**2.01 (0.04, 3.97)**	**0.045**	**2.24 (0.41, 4.07)**	**0.017**
Calcium/vitamin D supplementation prior to admission	**5.10 (1.54, 8.67)**	**0.005**	**3.95 (0.42, 7.47)**	**0.028**
Multivitamin supplementation prior to admission	**4.84 (1.77, 7.91)**	**0.002**	2.96 (−0.08, 6.01)	0.057
Calcium, serum/plasma (mg/dL)	**3.16 (0.99, 5.33)**	**0.004**	**2.19 (0.14, 4.23)**	**0.036**
Phosphorous, serum/plasma (mg/dL)	−0.15 (−0.66, 0.37)	0.574	–	–

Boldface indicates *p* < 0.05

^a^ Unadjusted represents outputs from simple linear regression analyses with the listed independent variable and 25-hydroxyvitamin D level as the dependent variable

^b^ Independent variables significantly associated with 25-hydroxyvitamin D level in unadjusted analyses were included in a single multivariable model

## Discussion

This retrospective chart review found that nearly half of male and female adolescents and young adults with eating disorders requiring inpatient medical stabilization had vitamin D deficiency or insufficiency on admission. To our knowledge, this is the first study to report vitamin D levels specifically in a male eating disorder sample and to examine sex differences in 25-hydroxyvitamin D concentrations. Although the prevalence of vitamin D deficiency and insufficiency did not differ according to sex, the prevalence of vitamin D deficiency was equally severe in the male population. Nearly half of the male eating disorder participants had vitamin D insufficiency, with 18% having levels consistent with deficiency (< 20 ng/mL) per the Endocrine Society [32] and the Society for Adolescent Health and Medicine [33]. A significant percentage of male participants had severe vitamin D deficiency (< 12 ng/mL), notably higher than that of female participants. A prior study of healthy young adults found that male sex was associated with vitamin D deficiency (< 20 ng/mL) [39]. This finding underscores the critical need to examine sex-specific nutritional deficiencies in adolescents and young adults with eating disorders.

Despite a large proportion of males presenting with vitamin D insufficiency or deficiency (44%), only 3% were receiving calcium or vitamin D-specific supplementation. Although muscle-building supplements such as protein or creatine are common among adolescent and young adult males [40–43], these do not provide micronutrients or vitamin D. While 9% of participants reported taking multivitamins, the calcium and vitamin D content of those supplements is unclear given the heterogeneity in the formulation of over-the-counter multivitamins. The lack of appropriate supplementation prior to hospital admission highlights the current lack of awareness of nutritional complications of EDs in males. Although 25-hydroxyvitamin D level was not significantly associated with sex, higher 25-hydroxyvitamin D levels were associated with prior calcium and vitamin D supplementation, emphasizing the importance of early monitoring and treatment of low vitamin D levels with supplementation.

Previous studies assessing vitamin D status in adolescents with EDs revealed inconsistent results, though a 2014 retrospective cohort by Modan-Moses demonstrated an even higher percentage of patients with vitamin D levels < 20 ng/ml as compared to our cohort (31% vs. our 12%) [44]. No difference in vitamin D levels were found between ED diagnoses, as in our sample, though none of the patients included in the 2014 study reported prior vitamin D supplementation. Only 45% of adolescents with anorexia nervosa consume the recommended daily allowance of vitamin D in their diet while only 25% consume the recommended daily allowance of calcium in their diet [45]. Both calcium and vitamin D have beneficial effects on bone mineralization, and vitamin D increases gut absorption of calcium [46, 47]. Recognizing nutrient deficiencies and/or providing prophylactic supplementation in patients with eating disorders may prevent further medical and psychological adverse outcomes.

In addition to calcium and vitamin D, several other factors have been found to be associated with bone mineral density in both males in females with eating disorders, though data are more limited in males [16, 46]. Suppression of the hypothalamic-pituitary-gonadal axis can occur in states of low energy availability including eating disorders, leading to lower estrogen and testosterone, which are critical for bone accrual in adolescence [46]. Anorexia nervosa is associated with reductions in insulin-like growth factor 1 (IGF1) and growth hormone, which are bone anabolic and facilitate periosteal bone apposition [46, 48]. The hypercortisolemia seen in anorexia nervosa stimulates osteoclasts, causes further dysregulation of the growth hormone-IGF1 axis, and impairs renal and gastrointestinal absorption of calcium, further yielding alterations in bone metabolism [49]. Overall, lower body mass index, as a proxy for greater degree of malnutrition [31], is associated with lower bone mineral density, greater fracture risk, and other medical complications among males and females with eating disorders [16, 17, 27, 28, 47, 48, 50–52].

Limitations of this study include its retrospective and observational nature, which precludes causal inferences. Findings are from a tertiary care hospital in Northern California and may not be generalizable to other populations, since geography (i.e., latitude) and amount of sun exposure can affect vitamin D levels. For instance, young adults living in Pennsylvania have significantly lower 25-hydroxyvitamin D levels than young adults living in Florida [39]. However, season and latitude only explain one fifth of the variability in 25-hydroxyvitamin D levels [53], highlighting the importance of behavioral factors. We did not collect data on specific activities that provide sun exposure, nor did we have a control population (e.g., adolescents and young adults hospitalized for other reasons). Selection bias is a possibility; however, there were no significant differences in demographic or anthropometric data between those included in the study and the 36 participants (including 4 male participants) excluded due to eligibility criteria except that those excluded were more likely to be non-White (see Additional file 1: Table 1). Given that non-White race was associated with lower 25-hydroxyvitamin D levels, we may have slightly underestimated rates of 25-hydroxyvitamin D insufficiency or deficiency. Strengths of this study include a robust participant sample gathered over eight years, whose clinical information was gathered by specialized eating disorder treatment teams. It is noteworthy that over 16% of sample participants were male, more than triple the proportion of males included in prior studies [1–3]. To our knowledge, this represents the first study to examine vitamin D deficiency in male populations with eating disorders.

## Conclusion

The vast majority of research on the medical complications of eating disorders has been conducted in exclusively or primarily female samples [50]. Because eating disorders and their medical complications affect both males and females, there is a critical need for studies to examine medical complications in male samples to inform sex-specific clinical practice guidelines [54]. Clinical guidance from the Society for Adolescent Health and Medicine’s Position Paper on the Medical Management of Restrictive Eating Disorders [31] describes certain aspects of medical management that only applies to females [55], although many medical complications are equally severe in males as in females with eating disorders [28]. Similarly, this study demonstrates that vitamin D deficiency and/or insufficiency in hospitalized male patients with eating disorders is common; however, few receive calcium or vitamin D supplementation prior to admission. Medical providers should consider assessing for vitamin D deficiency, especially in male adolescent and young adult populations with eating disorders.

## Supplementary Information

**Additional file 1: Table 1.** Comparison of included vs excluded participants. **Table 2.** 25-hydroxyvitamin D levels of adolescents and young adults hospitalized for restrictive eating disorders by sex, excluding patients with active calcium or vitamin D supplementation on admission. **Table 3.** Factors associated with 25-hydroxyvitamin D level excluding patients with active calcium or vitamin D supplementation on admission, linear regression analysis.

## Data Availability

The data that support the findings of this study are available on request from the corresponding author, JN. The data are not publicly available due to confidentiality restrictions e.g. their containing information that could compromise the privacy of research participants.

## Abbreviations

AN: Anorexia nervosa
CLIA: Clinical Laboratory Improvement Amendments
EDNOS: Eating disorder not otherwise specified
EMR: Electronic medical records
