# Supplementary material for: Assessment of vitamin D among male adolescents and young adults hospitalized with eating disorders
Source: J Eat Disord. 2022 Jul 18;10:104. doi: 10.1186/s40337-022-00627-5 (PMC9295348; doi:10.1186/s40337-022-00627-5)
Supplement: Supplementary file 1 — Additional file 1: Table 1. Comparison of included vs excluded participants. Table 2. 25-hydroxyvitamin D levels of adolescents and young adults hospitalized for restrictive eating disorders by sex, excluding patients with active calcium or vitamin D supplementation on admission. Table 3. Factors associated with 25-hydroxyvitamin D level excluding patients with active calcium or vitamin D supplementation on admission, linear regression analysis. [file 40337_2022_627_MOESM1_ESM.docx]

| Additional file 1: Table 1. Comparison of included vs excluded participants^a^ | | | |
| --- | --- | --- | --- |
| Sociodemographic characteristics | Included (n = 565) | Excluded (n = 36) | p |
| Sex, n (%) |  |  | 0.490 |
| Female | 472 (83.54%) | 32 (88.89%) |  |
| Male | 93 (16.46%) | 4 (11.11%) |  |
| Age, years, mean (sd) | 15.99 | 16.03 | 0.943 |
| Race/ethnicity, n (%) |  |  | **0.004** |
| Non-Hispanic white | 343 (60.71%) | 13 (36.11%) |  |
| All other race/ethnicities | 222 (39.29%) | 23 (63.89%) |  |
| Diagnosis, n (%) |  |  | 0.960 |
| Anorexia nervosa | 332 (58.76%) | 21 (58.33%) |  |
| All other diagnoses | 233 (41.24%) | 15 (41.67%) |  |
| BMI, kg/m^2^, mean (sd) | 17.55 | 17.93 | 0.462 |
| ^a^ Participants were excluded due to missing 25-hydroxyvitamin D levels. | | | |
| P-value is for t-tests for continuous variables (age, BMI), Chi-squared tests for categorical variables (race/ethnicity, diagnosis), and Fisher’s exact tests for categorical variables with five or fewer participants in a cell (sex). | | | |

| Additional file 1: Table 2. 25-hydroxyvitamin D levels of adolescents and young adults hospitalized for restrictive eating disorders by sex, excluding patients with active calcium or vitamin D supplementation on admission^a^ | | | | |
| --- | --- | --- | --- | --- |
|  |  | Sex | |  |
| Characteristic | Total (N=519) | Male (N = 90) | Female (N = 429) | p ^b^ |
| 25-hydroxyvitamin D (ng/mL), mean (sd) | 31.74 ± 11.85 | 29.82 ± 11.79 | 32.15 ± 11.84 | 0.091 |
| 25-hydroxyvitamin D categories, n (%) |  |  |  |  |
| Insufficiency (<30 ng/mL) | 233 (44.89) | 41 (45.56) | 192 (44.76) | 0.89 |
| Deficiency (<20 ng/mL) | 67 (12.91) | 17 (18.89) | 50 (11.66) | 0.063 |
| Severe deficiency (<12 ng/mL) | 17 (3.28) | 8 (8.89) | 9 (2.10) | **0.001** |
| ^a^ Table values are mean ± SD for continuous variables and n (column %) for categorical variables. | | | | |
| ^b^ P-value is for t-tests for continuous variables or Pearson's chi square tests for categorical variables, respectively. | | | | |

| Additional file 1: Table 3. Factors associated with 25-hydroxyvitamin D level excluding patients with active calcium or vitamin D supplementation on admission, linear regression analysis | | | | |
| --- | --- | --- | --- | --- |
|  | Unadjusted | | Adjusted | |
| Independent variables | B (95% CI)^a^ | p | B (95% CI)^b^ | p |
| Male sex | -2.32 (-5.02, 0.37) | 0.091 | -- | -- |
| Age, years | 0.04 (-0.33, 0.40) | 0.847 | -- | -- |
| White (vs. non-White) | **8.09 (6.12, 10.07)** | **<0.001** | **7.59 (5.62, 9.56)** | **<0.001** |
| Anorexia nervosa (vs. non-anorexia Nervosa) | **2.54 (0.47, 4.60)** | **0.016** | 1.91 (-0.12, 3.94) | 0.065 |
| Percent median BMI | **-8.11 (-15.32, -0.91)** | **0.027** | -2.85 (-9.92, 4.22) | 0.429 |
| Summer (vs. winter) | 1.91 (-0.14, 3.96) | 0.068 | **2.12 (0.20, 4.04)** | **0.031** |
| Multivitamin supplementation prior to admission | **4.26 (0.57, 7.95)** | **0.024** | 2.96 (-0.51, 6.43) | 0.094 |
| Calcium, serum/plasma (mg/dL) | **3.48 (1.26, 5.69)** | **0.002** | **2.73 (0.64, 4.83)** | **0.011** |
| Phosphorous, serum/plasma (mg/dL) | -0.14 (-0.66, 0.37) | 0.586 | -- | -- |
| Boldface indicates p<0.05 |  |  |  |  |
| ^a^ Unadjusted represents outputs from simple linear regression analyses with the listed independent variable and 25-hydroxyvitamin D level as the dependent variable. | | | | |
| ^b^ Independent variables significantly associated with 25-hydroxyvitamin D level in unadjusted analyses were included in a single multivariable model. We included season (April through September vs. October through March) given that season was significantly associated with 25-hydroxyvitamin D level in the original analysis. | | | | |
